# A mouse model for ulcerative colitis based on NOD-*scid* IL2R γ^null^ mice reconstituted with peripheral blood mononuclear cells from affected individuals

**DOI:** 10.1242/dmm.025452

**Published:** 2016-09-01

**Authors:** Pia Palamides, Henrika Jodeleit, Michael Föhlinger, Florian Beigel, Nadja Herbach, Thomas Mueller, Eckhard Wolf, Matthias Siebeck, Roswitha Gropp

**Affiliations:** 1 Institute of Molecular Animal Breeding and Biotechnology, Gene Center, LMU Munich, Munich 81377, Germany; 2 Laboratory for Functional Genome Analysis (LAFUGA), Gene Center, LMU Munich, Munich 81377, Germany; 3 Department of General- Visceral-, and Transplantation Surgery, Hospital of the University of Munich, Nussbaumstr. 20, Munich 80336, Germany; 4 Department of Medicine II-Grosshadern, Ludwig Maximilians University, Munich, Germany; 5 Institute of Veterinary Pathology, Ludwig Maximilians University, Munich, Germany; 6 Julius von Sachs Institute, University of Würzburg, Würzburg 97082, Germany

**Keywords:** Ulcerative colitis, NSG mice, Infliximab, Pitrakinra

## Abstract

Animal models reflective of ulcerative colitis (UC) remain a major challenge, and yet are crucial to understand mechanisms underlying the onset of disease and inflammatory characteristics of relapses and remission. Mouse models in which colitis-like symptoms are induced through challenge with toxins such as oxazolone, dextran sodium sulfate (DSS) or 2,4,6-trinitrobenzenesulfonic acid (TNBS) have been instrumental in understanding the inflammatory processes of UC. However, these neither reflect the heterogeneous symptoms observed in the UC-affected population nor can they be used to test the efficacy of inhibitors developed against human targets where high sequence and structural similarity of the respective ligands is lacking. In an attempt to overcome these problems, we have developed a mouse model that relies on NOD-*scid* IL2R γ^null^ mice reconstituted with peripheral blood mononuclear cells derived from UC-affected individuals. Upon challenge with ethanol, mice developed colitis-like symptoms and changes in the colon architecture, characterized by influx of inflammatory cells, edema, crypt loss, crypt abscesses and epithelial hyperplasia, as previously observed in immune-competent mice. TARC, TGFβ1 and HGF expression increased in distal parts of the colon. Analysis of human leucocytes isolated from mouse spleen revealed an increase in frequencies of CD1a+, CD64+, CD163+ and TSLPR+ CD14+ monocytes, and antigen-experienced CD44+ CD4+ and CD8+ T-cells in response to ethanol. Analysis of human leucocytes from the colon of challenged mice identified CD14+ monocytes and CD11b+ monocytes as the predominant populations. Quantitative real-time PCR (RT-PCR) analysis from distal parts of the colon indicated that IFNγ might be one of the cytokines driving inflammation. Treatment with infliximab ameliorated symptoms and pathological manifestations, whereas pitrakinra had no therapeutic benefit. Thus, this model is partially reflective of the human disease and might help to increase the translation of animal and clinical studies.

## Abstract (precis)

**Summary:** The phenotype and colitis-like symptoms induced in NSG mice reconstituted with PBMCs derived from ulcerative-colitis-affected donors reflect the human disease.

## INTRODUCTION

Animal models present one of the biggest scientific challenges in exploring the etiology of complex inflammatory diseases. For practical reasons, mice are the preferred animals in which cases of ulcerative colitis (UC) are usually induced with toxins such as oxazolone, dextran sodium sulphate (DSS) or 2,4,6-trinitrobenzenesulfonic acid (TNBS), leading to the development of colitis-like symptoms (Kiesler et al., 2015). However, these models differ substantially from the human disease because they poorly reflect the pathophysiological mechanisms of a genetically heterogeneous population of affected individuals that are often diseased for decades. In addition, they cannot be used when species-specific responses are involved, or when high sequence and/or structural similarity between inhibitors and ligands across species is required in order to interact with receptors of interest. Therefore, we have developed a model that is based on immunocompromised NOD-*scid* IL2R γ^null^ (NSG) mice reconstituted with peripheral blood mononuclear cells (PBMCs) derived from UC-affected individuals (Nolte et al., 2013a). In this model, UC-like symptoms were induced through rectal challenge with oxazolone. Unexpectedly, similar, albeit milder, effects were observed with ethanol as the solvent for oxazolone when a UC individual served as donor. This observation prompted us to assume that the inflammatory cells of UC individuals increase the susceptibility of mice to develop colitis and thus might be more reflective of the human disease. Here, we report that NSG mice reconstituted with PBMCs derived from UC individuals developed similar symptoms to those previously observed in oxazolone-challenged mice (Nolte et al., 2013a). Challenge with ethanol resulted in a mixed infiltrate of immune cells into the lamina propria that comprised CD4+, CD8+ T-cells, CD11b+ macrophages and CD14+ monocytes. Colon architecture was characterized by the development of edema, fibrosis, crypt abscesses and hemorrhage. The severity of disease symptoms and pathological manifestations were donor dependent. The response to ethanol resulted in an increase of subtypes of CD14+ monocytes to include CD64-, CD163-, TSLPR- and CD1a-expressing monocytes, as well as antigen-experienced CD4+ splenic human leucocytes. Treatment with infliximab ameliorated the symptoms and pathological manifestations, and resulted in a similar immunological signature to that observed in UC individuals treated with infliximab (Ulrich Mansmann and our unpublished data), characterized by increased levels of fibrosis, and reduced HGF and TARC expression. Conversely, treatment with the IL-4Rα inhibitor pitrakinra had no therapeutic effect but exacerbated symptoms and pathological manifestations. Treatment resulted in an increase of CD8+ cells and central memory CD8+ cells in splenic human leucocytes and decreased fibrosis, suggesting that the suppression of the T-helper cell 2 (Th2) inflammatory arm favors an auto-immune reaction to increase damage to the mucosa.

## RESULTS

### Characterization of inflammatory response in ethanol-challenged mice

**Fig. 1.**
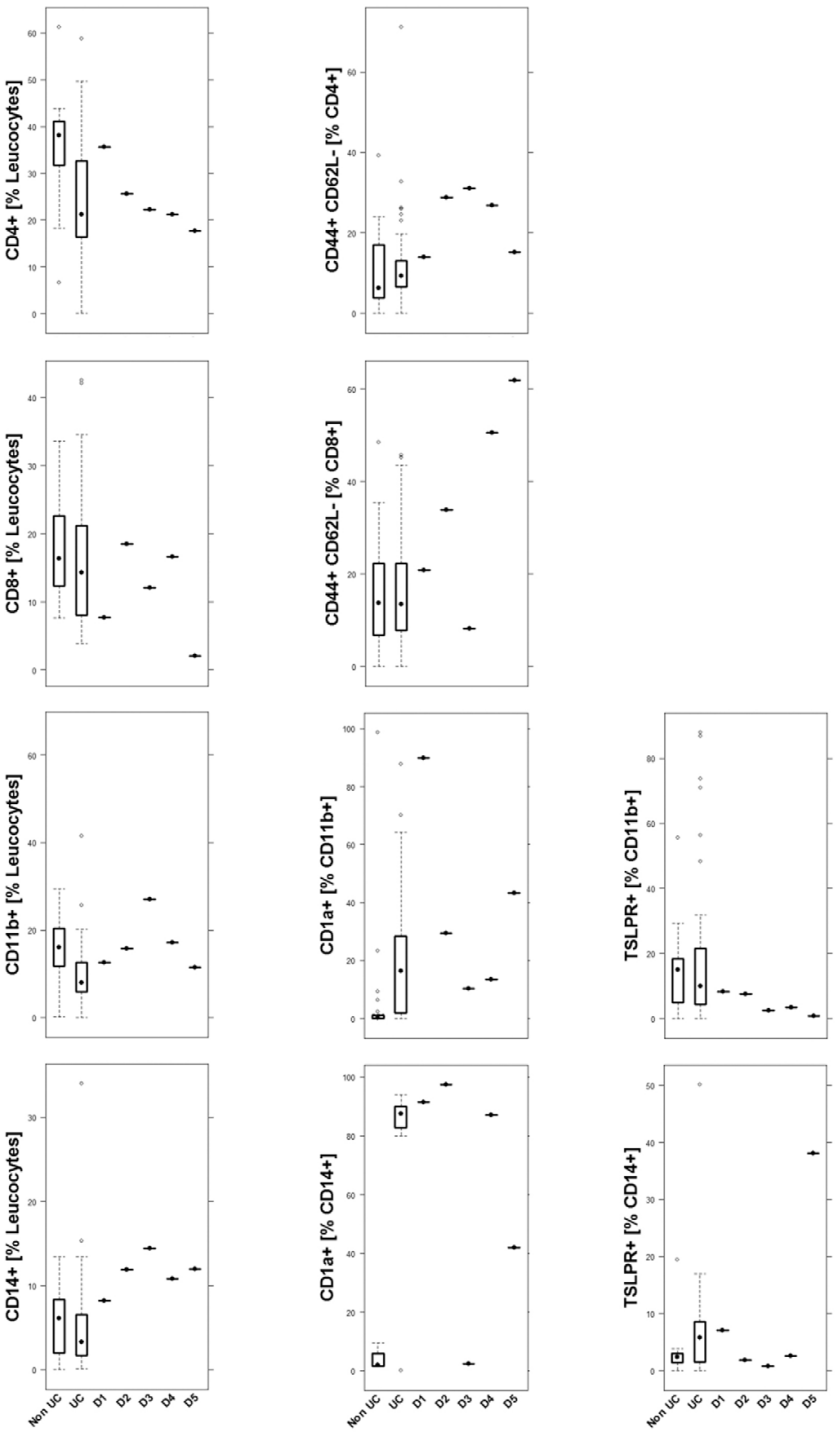
**The immunological profile of donor PMBCs selected to reconstitute mice.** Boxplot analysis of isolated PBMCs that were subjected to flow cytometric analysis. Sample size: CD4+, CD4+ CD44+ CD62L−, CD8+, CD8+ CD44+ CD62L−, CD14+ non-UC *n*=30, UC *n*=40; CD11b+, CD11b+ CD1a+ non-UC *n*=31, UC *n*=40; CD11b+ TSLPR+, CD14+ TSLPR+ non-UC *n*=15, UC *n*=40; CD14+ CD1a+ non-UC *n*=9, UC *n*=27. Labels given on *x*-axes on the bottom row apply to all charts. Boxes represent upper and lower quartiles, whiskers represent variability and outliers are plotted as individual points. Lines represent values without variability. D, donor.

In order to gain a better understanding of the inflammatory responses evoked by the challenge with ethanol in NSG mice reconstituted with PBMCs derived from UC-affected individuals and to elucidate whether this model is reflective of the human disease, the response to challenge was analyzed with regard to the development of a clinical and histological score, macroscopic changes of the colon, the frequency of leucocytes isolated from the spleen and colon, and cytokine and growth factor expression in the colon. Mice were reconstituted with 3×10^6^-4×10^6^ human PBMCs from UC individuals (*n*=5) as described in Materials and Methods. All donors had clinical activity scores between 5 and 10, as determined by the simple clinical colitis activity indices (SCCAI), and were considered as being in relapse. Two donors were treated with infliximab, one with mesalazine and two were untreated. To determine the profile of cells that had been injected into the mice, PBMCs were subjected to flow cytometric analysis before injection. As shown in Fig. 1, all donors exhibited high levels of antigen-experienced CD4+ T-cells, CD14+ monocytes and CD11b+ macrophages as compared to non-UC subjects. This also applied to effector memory CD8+ T-cells, with the exception of donor 3. Donor 3 also differed from the other donors with regard to CD1a+ monocytes, where all other donors displayed elevated levels as compared to non-UC subjects. The highest variability between donors was observed in CD1a-expressing CD11b+ macrophages.

**Fig. 2.**
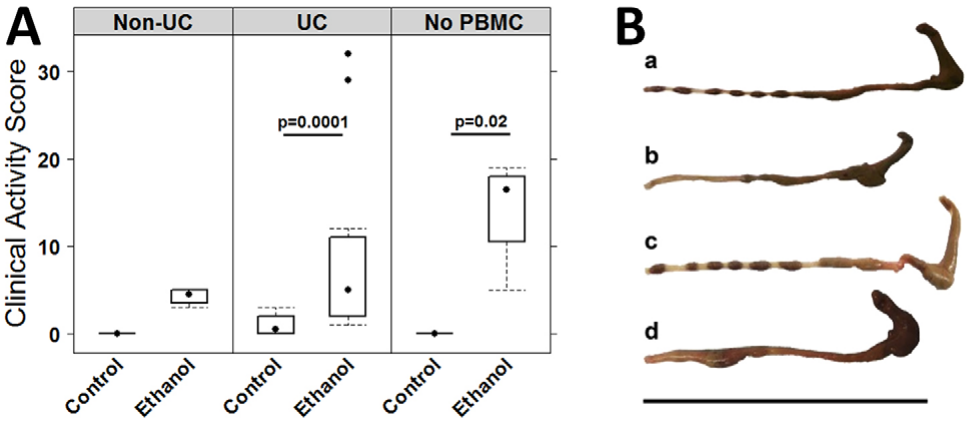
**Challenge with ethanol results in development of colitis-like symptoms in NSG mice that had been engrafted with PMBCs derived from a UC individual.** (A) Clinical activity score depicted as a boxplot diagram. Sample sizes: mice reconstituted with PBMCs from a non-UC donor, unchallenged control, *n*=4; challenged (ethanol) control, *n*=4. Mice reconstituted with PBMCs from a UC donor, unchallenged control, *n*=20; challenged, *n*=20. Non-reconstituted mice (no PMBC), unchallenged, *n*=4; challenged, *n*=4. For comparison of unchallenged control versus challenged, a Student's *t*-test was performed. Boxes represent upper and lower quartiles, whiskers represent variability and outliers are plotted as individual points. Lines represent values without variability. (B) Macrophotographs of colons at autopsy of NSG mice that had been engrafted with PBMCs from a UC donor. (a) unchallenged control, (b) challenged with ethanol, (c) engrafted with PBMCs from a non-UC donor challenged with ethanol, (d) non-engrafted challenged with ethanol. Scale bar: 10 cm.

Seven days post reconstitution, the mice were divided into two groups: one was left unchallenged and the other was challenged through rectal application of ethanol. Each group contained four animals. As we had previously observed high toxicity of oxazolone in non-reconstituted mice, an additional control comprising non-reconstituted mice was added (Nolte et al., 2013a). In addition, to support our previous observations that the histological score was highest when PBMCs from a donor with UC was used for reconstitution, a group of mice was added that had been reconstituted with PBMCs from a non-UC donor. On day seven, mice were pre-sensitized with rectal application of 10% ethanol, followed by rectal application of 50% ethanol at days 15 and 18. The onset of the disease was monitored by measuring body weight, and visual inspection of stools and mice. Symptoms were classified according to a clinical activity score as described in Materials and Methods. Upon challenge with ethanol, stools of mice reconstituted with PBMCs from UC donors became soft or liquid, the animals lost weight and the activity was reduced. Unchallenged control animals displayed no symptoms. Symptoms peaked at day 16, and challenged animals recovered two days post challenge. All animals except one survived. The development of symptoms was reflected in the clinical activity score of the challenged group, which was significantly higher as compared to that of the unchallenged group (Fig. 2A). (For complete data set see Table S1.) As observed in previous experiments, unchallenged control animals remained unaffected. We observed a high variability between different donors. Mice reconstituted with a non-UC donor also developed a higher clinical score, but unlike the group that had been reconstituted with PBMCs from UC donors, the stool consistency was unaffected. As previously observed in oxazolone-challenged mice, challenge with ethanol in the absence of PBMCs was highly toxic, and three animals had to be euthanatized before the end of the study. These animals also displayed no diarrhea.

On day 21, mice were killed, the colon was visually inspected, and colon samples from the distal part of the colon were collected for histological and mRNA expression analysis, and leucocytes were isolated from spleen and colon. Visual inspection of the colon corroborated the observed clinical scores.

**Fig. 3.**
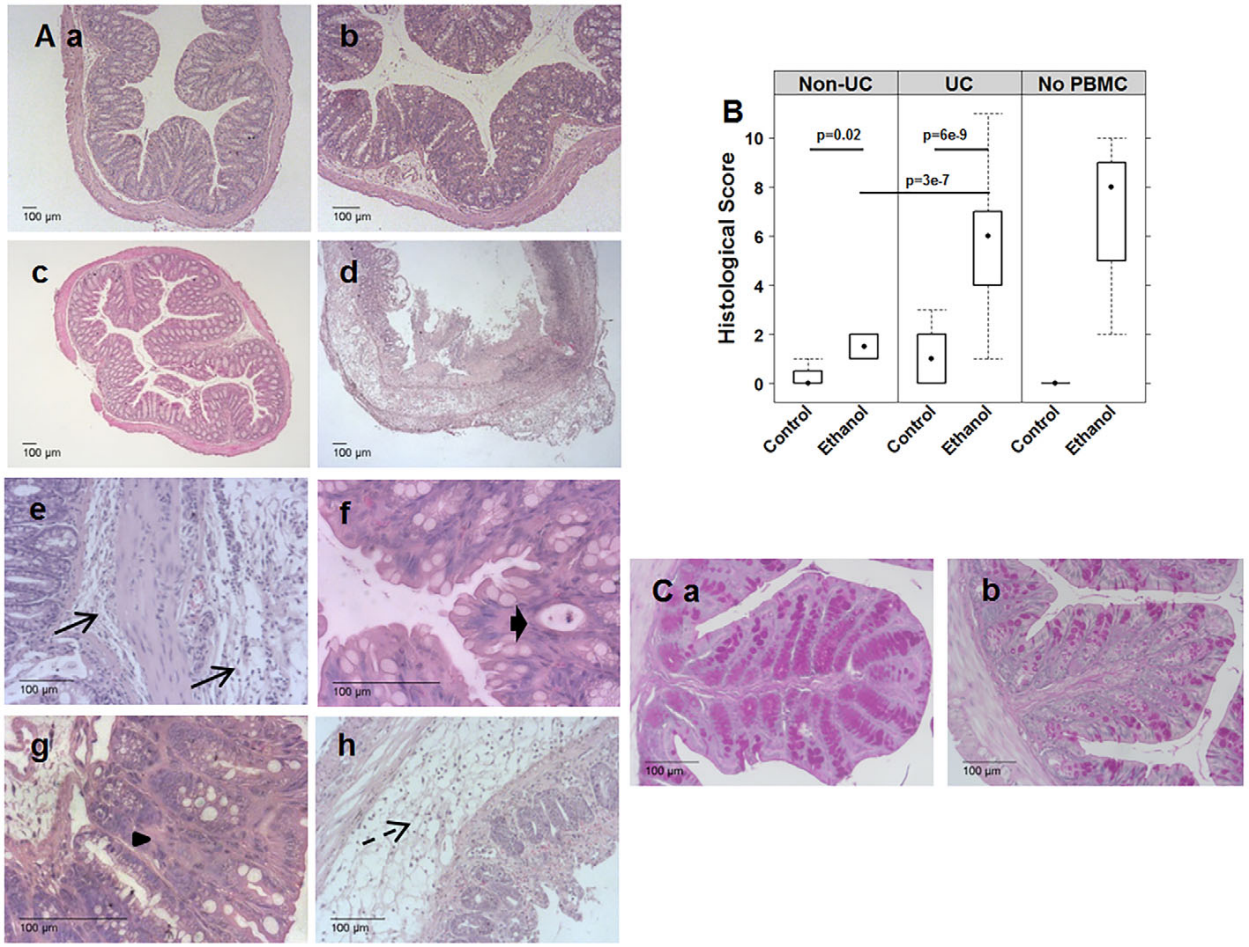
**Morphological changes in response to ethanol were dependent on the immunological background of the donor and the presence of PBMCs.** Challenge of NSG mice with 10% ethanol at day 7, and 50% ethanol at days 15 and 18 that had been engrafted with PBMCs from a UC donor resulted in edema, crypt abscesses, crypt loss, fibrosis, epithelial erosions and infiltration of inflammatory cells into the submucosa and lamina propria. Challenge of NSG mice engrafted with PBMCs from a non-UC donor had no effect, and challenge of non-engrafted NSG mice resulted in influx of neutrophils, crypt loss and edema. (A) Photomicrographs of stained H&E paraffin sections of distal parts of the colons of NSG mice that had been engrafted with PBMCs derived from UC donors. (a) Engrafted with PBMCs from a UC donor, unchallenged control. (b,e,f,g,h) engrafted with PBMCs from a UC donor and challenged with ethanol. Arrow indicates influx of inflammatory cells; bold arrow, crypt abscesses; arrowhead, fibrosis. (c) Engrafted with PBMCs from a non-UC donor, challenged with ethanol. (d) Non-engrafted, challenged with ethanol. (B) Histological alterations were classified according to a histological score and depicted as a boxplot diagram. Non-UC sample sizes, unchallenged control, *n*=4; challenged with ethanol, *n*=4. UC: experiments were performed with five different donors. Sample sizes: unchallenged control, *n*=20; challenged with ethanol, *n*=20. No PBMC sample sizes: unchallenged, *n*=1; challenged, *n*=4. A two-sided Student's *t*-test and confidence level=0.95 was used to compare groups. Boxes represent upper and lower quartiles, whiskers represent variability and outliers are plotted as individual points. Lines represent values without variability. (C) Photomicrographs of PAS-stained paraffin sections of distal parts of the colon. (a) BALB/c mouse; (b) non-engrafted NSG mouse.

As shown in Fig. 2B, challenge with ethanol affected mice differently, dependent on the presence of PBMCs and the immunological background of the donor. When mice that had been reconstituted with PBMCs from a UC donor were challenged with ethanol, the colon had been emptied of stool and was dilated, and in some cases, displayed hyperemia (Fig. 2Bb). In contrast, colons of control animals displayed no signs of diarrhea or inflammation as indicated by solid and evenly dispersed stool pellets (Fig. 2Ba). Colons of mice reconstituted with PBMCs from a healthy donor displayed no signs of inflammation and were indistinguishable from colons of the control group of unchallenged mice (Fig. 2Bc). In contrast, in the absence of PBMCs, challenge with ethanol resulted in severe damage of the colon (Fig. 2Bd). Exactly in the region where the concentration of ethanol was supposedly high, the colon was constipated, indicating loss of peristaltic movement. The same observations were made in a previous study (Nolte et al., 2013a) in which oxazolone was used to induce colitis; however, in that case, the toxicity of oxazolone was even higher. Histological analysis further corroborated these observations. Analysis of hematoxylin and eosin (H&E)-stained sections from the distal part of the colon of mice that had been reconstituted with PBMCs from a UC donor revealed that the response to challenge with ethanol resulted in morphological changes of the colon architecture that were characterized by edema, influx of a mixed infiltrate of leucocytes into the mucosa and submucosa, focal fibrosis of the mucosa, epithelial erosions, single crypt abscesses and hyperemia. In addition, increased basophilia at the base of the crypts indicated epithelial proliferation (Fig. 3Ab). Hardly any morphological changes were observed when mice were reconstituted with PBMCs from a healthy donor (Fig. 3Ac).

In the absence of PBMCs, challenge with ethanol resulted in severe damage of the mucosa, similar to that observed in mice challenged with oxazolone. Mucosal damage was accompanied by strong influx of neutrophils (Fig. 3Ad).

The morphological changes were classified according to a histological score as described in Materials and Methods. As observed with the clinical activity score, the degree of pathological manifestation varied between experiments and indicated a donor-to-donor variability. As shown in Fig. 3B, the histological score in all challenged groups was significantly higher as compared to the control groups (for complete data set see Table S1). The histological score was lowest when mice were reconstituted with PBMCs from a non-UC donor, and it was highest in the absence of PBMCs. In order to examine whether impaired mucus production could lead to toxic responses to ethanol, periodic-acid–Schiff staining (PAS) was performed. As shown in Fig. 3C, in sections of wild-type BALB/c mice, the number of PAS-stained cells was higher, and the distribution of goblet cells more regular as compared to NSG mice.

To further characterize the inflammatory response, human leucocytes that had been isolated from murine spleens were subjected to flow cytometric analysis according to the cellular markers displayed in Table S5 (for gating strategy see Fig. S1).

**Fig. 4.**
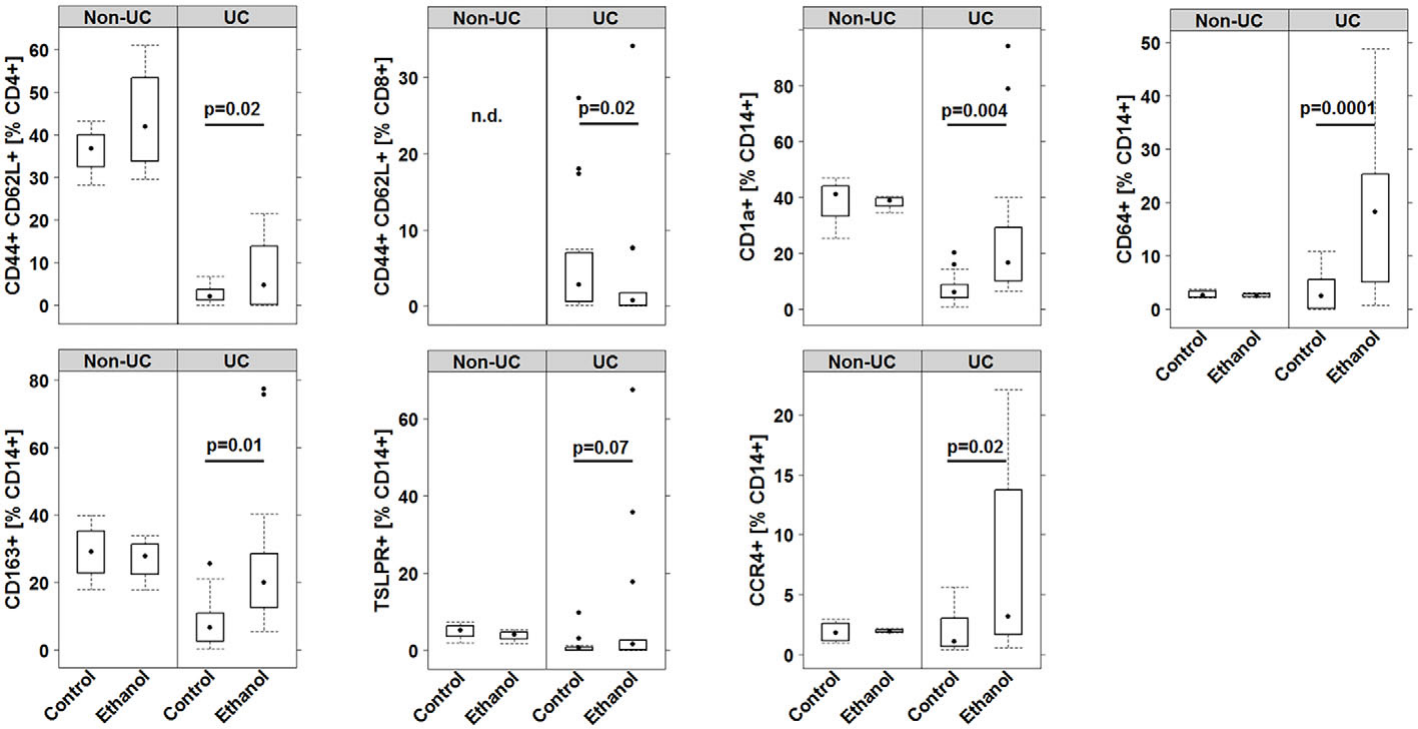
**Challenge with ethanol affected subgroups of human T-cells and CD14+ monocytes isolated from spleens of NSG mice that had been reconstituted with PBMCs from a UC-affected individual.** Boxplot analysis of human leucocytes isolated from spleens of mice and subjected to flow cytometry analysis for the indicated markers. Mice were challenged with 10% ethanol at day 7, and 50% ethanol at days 15 and 18. Experiments were performed with five different UC donors and one non-UC donor. Labels given on *x*-axes on the bottom row apply to all charts. (For sample sizes and complete data set see Table S1). For comparison of control versus challenged, a Student's *t*-test was performed. Boxes represent upper and lower quartiles, whiskers represent variability and outliers are plotted as individual points. Lines represent values without variability.

Human leucocytes that had been isolated from the spleen of mice reconstituted with PBMCs from a non-UC individual and a UC individual displayed different patterns in response to ethanol (Fig. 4). Frequencies of human leucocytes isolated from the spleen of mice that had been reconstituted with PBMCs from the non-UC donor did not change in response to ethanol, with the exception of antigen-experienced CD4+ T-cells. In addition, experienced CD8+ T-cells were not detected. In contrast, ethanol induced a change in frequencies of human leucocytes. Analysis of human CD45 revealed mean engraftment levels of 9.07%±9.25 leucocytes (mean±s.d.) in the control group and 15.46%±12.69 in the challenged group (for complete data set see Table S1). The increase just failed to reach significance (*P*=0.07). Challenge with ethanol resulted in a significant increase in antigen-experienced CD4+ T-cells, and subsets of CD14+ monocytes such as CD64+, CD163+, CCR4+, CD1a+ monocytes. TSLPR-expressing monocytes displayed a trend towards significance. In contrast, a significant decline in effector memory CD8+ cells was observed (for complete data set, please see Table S1). As observed in the clinical and histological scores, variability was high.

**Fig. 5.**
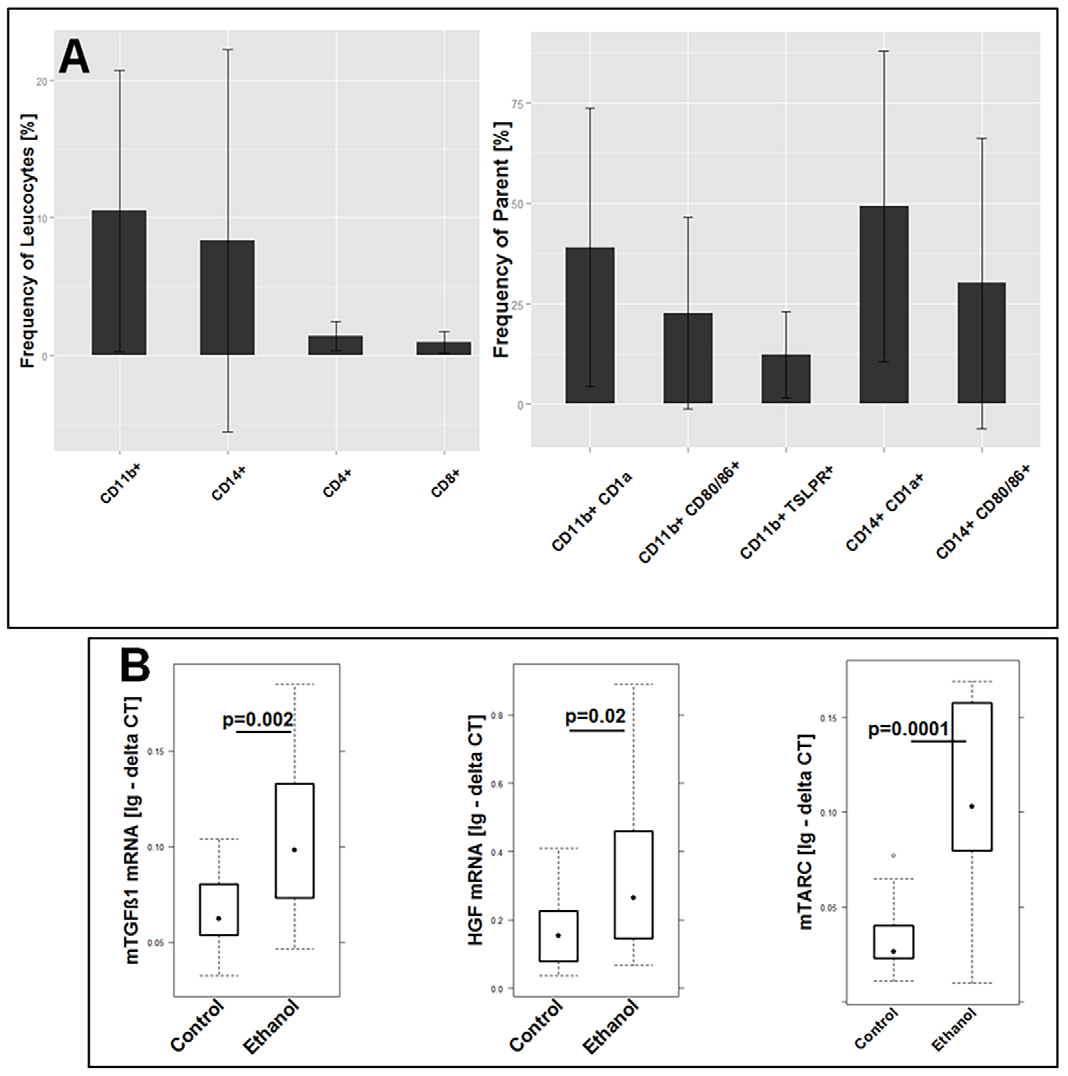
**Challenge with ethanol induces influx of inflammatory cells into the colon and increased expression of TGFβ1 and HGF in NSG mice that had been reconstituted with PBMCs from UC donors.** (A) Identification of T-cells, macrophages and monocyte populations. Human leucocytes were isolated from colons of mice that had been challenged with ethanol and were subjected to flow cytometry analysis. Frequency of CD11b+ macrophages, CD14+ monocytes, and CD4+ and CD8+ T-cells, and the frequency of subtypes of CD11b+ macrophages and CD14+ monocytes in the colon of challenged NSG mice. Sample size: *n*=6. Mean values are given, error bars are s.d. (B) Boxplot analysis of mRNA expression of mouse (m)TGFβ1, HGF and mouse (m)TARC in distal parts of the colon of NSG mice in response to challenge with 10% ethanol at day 7, and 50% ethanol at days 15 and 18. RNA was isolated from distal parts of the colon and subjected to RT-PCR analysis. Sample size: unchallenged control, *n*=16; challenged with ethanol, *n*=16. For comparison of control versus challenged, a Student's *t*-test and confidence level=0.95 was used (for complete data set see Table S1). Boxes represent upper and lower quartiles, whiskers represent variability and outliers are plotted as individual points. Lines represent values without variability. Ig - delta CT, logarithmic delta cycle threshold; parent, parent cell population that subgroups were gated from.

Previous analysis has identified human T-cells as infiltrating cells when oxazolone is used as toxic agent (Nolte et al., 2013a). In order to further analyze the infiltrating cells, human leucocytes were isolated from mice colons and subjected to flow cytometric analysis as described in Materials and Methods (for gating strategy see Fig. S2). Owing to the low frequency of leucocytes, colons from each group were pooled for this experiment. As shown in Fig. 5A, CD14+ monocytes and CD11b+ macrophages were the most abundant populations and exceeded CD4+ and CD8+ T-cells in number. The variability of frequencies was high and reflected the donor-to-donor variability. The most abundant subsets of monocytes and macrophages were those that expressed CD1a or TSLPR. In addition, mRNA expression of mouse TGFβ1, HGF and mouse TARC increased significantly upon ethanol challenge, as observed in UC individuals.

### Response to treatment with infliximab and pitrakinra

Inhibitors are valuable tools to characterize inflammatory responses in a complex setting that involves the cross-talk of various cell types. One of the most efficacious therapeutic for UC is the anti-TNFα monoclonal antibody infliximab, although initially it had not been considered as a therapeutic for UC. In contrast to Crohn's disease, inflammation in UC has not been thought to be driven by TNFα. Therefore, it is still a point of discussion how infliximab exerts its efficacy. As the IgG1 effector function of the antibody seems crucial, it has now been suggested that binding of infliximab to surface TNFα might activate complement and induce apoptosis in TNFα-bearing cells (Scallon et al., 1995). In light of this, it seemed an attractive idea to examine the efficacy of infliximab at a cellular level in our mouse model. NSG mice were reconstituted as described above in the previous experiment, this time, however, a third group was added, which was treated with intraperitoneal injection of infliximab on days 7, 14 and 17. The isotype monoclonal antibody served as an additional control. The experiment was performed with two different donors, and each group contained four animals. All mice were subjected to the same analysis as in the previous experiment.

**Fig. 6.**
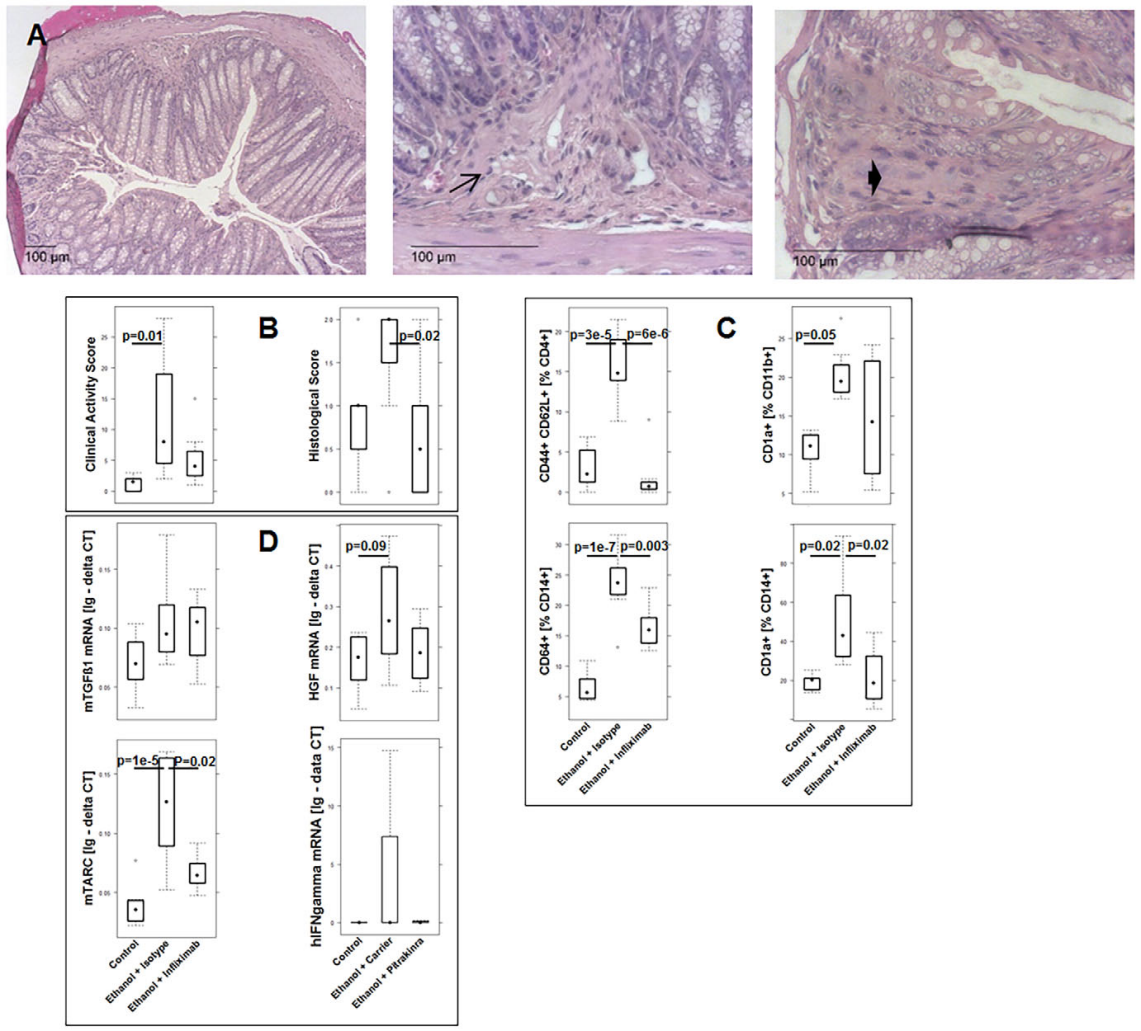
**The therapeutic effect of infliximab in reconstituted NSG mice challenged with ethanol.** (A) Photomicrographs of H&E-stained sections of distal parts of the colon from mice that had been challenged with 10% ethanol at day 7, and with 50% ethanol at days 15 and 18, and treated with infliximab at days 7, 14 and 17. Arrow indicates influx of inflammatory cells, bold arrow indicates fibrosis. (B) Boxplot analysis of the clinical activity and histological score. (C) Boxplot analysis of the frequency of human leucocytes isolated from spleen of NSG mice that had been treated as described in A. Quantification was performed using flow cytometry. (D) Boxplot analysis of mouse (m)TGFβ1 and mHGF expression in colon of NSG mice. RNA was isolated from distal parts of the colon and subjected to RT-PCR analysis. Experiments were performed with PBMCs from two different donors. Sample sizes: unchallenged control. *n*=8; challenged with ethanol and treated with isotype control, *n*=8; challenged with ethanol and treated with infliximab, *n*=8. For comparison of groups, ANOVA followed by Tukey's HSD was conducted. Labels given on *x*-axes on the bottom row apply to all charts. Boxes represent upper and lower quartiles, whiskers represent variability and outliers are plotted as individual points. Lines represent values without variability. Ig - delta CT, logarithmic delta cycle threshold.

As shown in Fig. 6, mice responded to treatment with infliximab (for complete data set see Table S2). Visual inspections of histological sections from distal parts of the colon revealed an influx of inflammatory cells into the lamina propria, however, at a reduced level (Fig. 6A) as compared to that in the ethanol-challenged group (Fig. 3Ab-Ah). Ethanol-induced fibrosis did not seem to be affected by infliximab. The clinical activity score almost returned to normal values, and this was reflected in the histological score (Fig. 6B). Treatment with infliximab affected human leucocytes that had been isolated from the spleen. Frequencies of antigen-experienced CD4+ T-cells and CD1a+ CD11b+ macrophages, as well as of CD64+ and CD1a+ CD14+ monocytes, increased in response to ethanol and declined when mice were treated with infliximab (Fig. 6C). Finally, mouse TGFβ1 expression was unaffected in contrast to that of HGF and mouse TARC (Fig. 6D). This result is consistent with data obtained from UC individuals. Here, treatment with blockers of TNFα resulted in increased expression of TGFβ1 whereas HGF and TARC expression levels decreased (Ulrich Mansmann and our unpublished data). In mice reconstituted with PBMCs from donor 5, human IFNγ was detected. The level increased in response to challenge with ethanol and decreased when mice were treated with infliximab.

IL-4 and IL-13 are thought to play a crucial role in Th2-characterized inflammatory diseases such as asthma and atopic dermatitis. Both exert their activities on the IL-4 receptor α1 (IL-4Rα1), which can either form a ternary complex type I with IL receptor common chain γ in the case of IL-4 or ternary complex type II with the IL-13α1 receptor in the case of IL-4 and IL-13. Activation of the type I complex leads to differentiation and proliferation of Th2 cells, as well as activation of the type II complex resulting in pathological manifestations such as fibrosis, mucus production and epithelial hyperplasia, and IgM-to-IgE switch (Mueller et al., 2002; LaPorte et al., 2008). As these pathological manifestations are hallmarks of asthma and atopic dermatitis, IL-4Rα1 has become a therapeutic target, and inhibition of IL-4Rα1 by the IL-4 variant pitrakinra or inhibitory monoclonal antibody dupilumab has shown efficacy in phase II clinical studies in asthma and atopic dermatitis, respectively (Wenzel et al., 2007; Beck et al., 2014). Because UC is also thought to be a Th2-characterized inflammation, albeit less clearly defined than in the two other diseases, we thought it an attractive idea to use pitrakinra in order to test for a potential therapeutic benefit. The target cells of pitrakinra in the mouse model are human leucocytes bearing the IL-4 receptor. This applies to subtypes of T-cells such as Th2 cells and subtypes of monocytes that have developed into M2 macrophages in the presence of IL-4 (Orme and Mohan, 2012). The experiment was conducted as the previous experiment above; however, in this experiment, mice were treated through intraperitoneal application of pitrakinra on days 7-9 and 14-21, as described in Materials and Methods. The experiment was performed with three different donors. Isotonic NaCl solution served as an additional control in the ethanol-challenged mice. The experiment was performed with three different donors, and each group contained four animals.

**Fig. 7.**
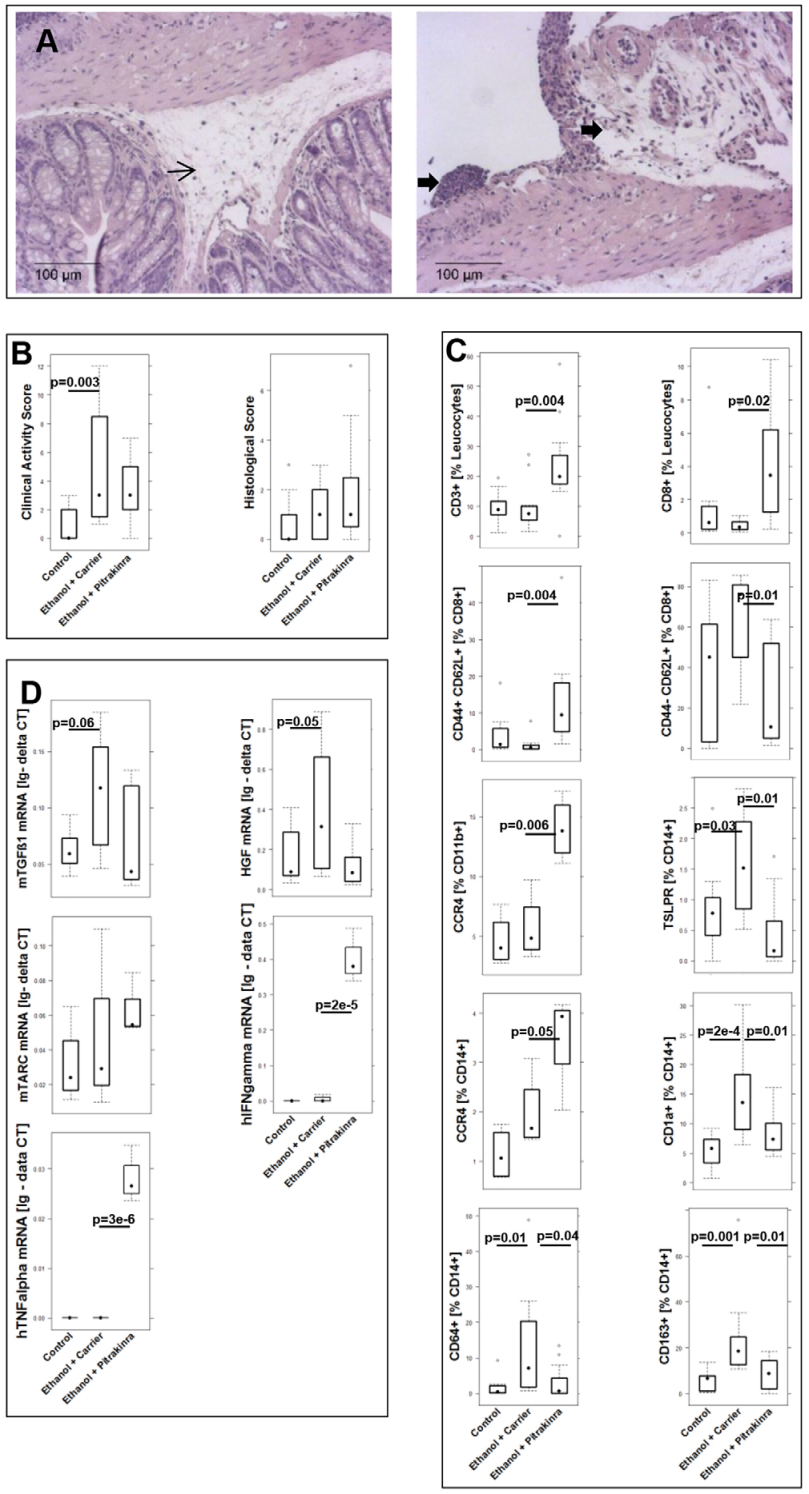
**Pitrakinra shows no therapeutic benefit in NSG mice challenged with ethanol.** (A) Photomicrographs of H&E-stained sections of distal parts of the colon from mice challenged with 10% ethanol at day 7, and with 50% ethanol at days 15 and 18, and treated with pitrakinra on days 7-9 and 14-21. Arrow indicates edema, and bold arrows indicate influx of inflammatory cells into the mesenterium. (B) Boxplot analysis of the clinical activity and histological scores. (C) Boxplot analysis of frequencies of the indicated human leucocytes that had been isolated from spleens and subjected to flow cytometry analysis. Experiments were performed with PBMCs from three different donors. Sample sizes: unchallenged control, *n*=12; challenged with ethanol and treated with solvent (carrier, isotonic sodium chloride solution), *n*=12; challenged with ethanol and treated with pitrakinra, *n*=11. (D) Boxplot analysis of mouse (m)TGFβ1, HGF and mouse (m)TARC mRNA expression in the colon of NSG mice. Human (h)TNFα and human (h)IFNγ mRNA was also measured. RNA was isolated from distal parts of the colon and subjected to RT-PCR analysis. Experiments were performed with PBMCs from two different donors. Sample sizes: unchallenged control, *n*=12; challenged with ethanol and treated with solvent, *n*=12; challenged with ethanol and treated with pitrakinra, *n*=11. (For complete data set, see Table S3.) For comparison of groups, ANOVA followed by Tukey's HSD was conducted. Labels given on *x*-axes on the bottom row apply to all charts. Boxes represent upper and lower quartiles, whiskers represent variability and outliers are plotted as individual points. Lines represent values without variability. Ig - delta CT, logarithmic delta cycle threshold.

Unexpectedly, treatment with pitrakinra had almost opposing effects to treatment with infliximab. Visual inspection of histological sections from distal parts of the colon revealed a strong influx of inflammatory cells in the lamina propria and mesenterium. Fibrosis seemed to not be as pronounced as in ethanol-challenged groups or as in ethanol-challenged groups treated with infliximab Fig. 7A. As shown in Fig. 7B, no improvement was observed with regard to the clinical activity and histological score. (For complete data set see Table S3.) The effect seen in human leucocytes that had been isolated from spleen of NSG mice might give an explanation to that observation. Frequencies of CD3+, CD8+, effector memory CD8+ and central memory CD8+ T-cells, and CCR4+ CD11b+ macrophages and CCR4+ CD14+ monocytes increased upon treatment with pitrakinra, suggesting that impairment of the Th2 inflammatory arm tipped the balance towards a T-helper cell 1 (Th1)-type inflammation. Conversely, an inhibitory effect was observed on the subset of monocytes that included TSLPR-, CD1a-, CD64- and CD163-expressing CD14+ monocytes, which were considered to be part of the ‘remodeling’ condition defined in humans with the exception of CD64+ CD14+ monocytes. In contrast to infliximab, TGFβ1 mRNA levels declined in response to treatment with pitrakinra, and mouse TARC mRNA expression levels increased. Increased TARC expression levels were found to be associated with an acute inflammatory condition in UC individuals (Ulrich Mansmann and our unpublished data). In addition, TARC expression was paralleled by an increase of CD14+ monocytes and CD11b+ macrophages bearing the TARC receptor CCR4. Furthermore, human IFNγ was increased in the pitrakinra-treated group, corroborating the shift towards a Th1 response. In this group, human TNFα could also be detected.

## DISCUSSION

The first aim of this study was to characterize the inflammatory response mounted in NSG mice that had been reconstituted with PBMCs derived from donors with UC upon challenge with ethanol. The second aim was to examine whether this model can be used for testing therapeutics addressing human target molecules, and the third was to elucidate whether, and to what degree, the evoked inflammation is reflective of the human disease.

### Characterization of the inflammation

As observed in conventional UC animal models that are based on immune-competent mice that have been exposed to toxic agents such as oxazolone, DSS or TNBS, challenge with ethanol resulted in weight loss, diarrhea, influx of leucocytes comprising lymphocytes, macrophages and neutrophils into the mucosa in NSG mice reconstituted with PBMCs from UC subjects. The inflammatory response resulted in an altered colon architecture characterized by edema, crypt loss, crypt abscesses, fibrosis and epithelial hyperplasia in reconstituted NSG mice. All these pathological manifestations were similar, albeit much less pronounced as compared to in the DSS mouse model, for example, and confirmed some of the previous results (Nolte et al., 2013a). The clinical activity and histological scores were highly donor dependent, suggesting that PBMCs from UC donors bear the memory of previous inflammations or possess a higher capacity to respond to challenge. This observation is also in agreement with previous data obtained in similar models (Nolte et al., 2013b; Zadeh-Khorasani et al., 2013). Analysis of human leucocytes that had been isolated from spleens of mice revealed that application of ethanol significantly affected antigen-experienced CD4+ and CD8+ T-cells and subtypes of CD14+ monocytes, indicating that CD14+ monocytes and T-cells are the main driver of this inflammatory response. Frequencies of three subtypes, namely CD64-, TSLPR- and CD1a-expressing monocytes have the capacity to relay inflammatory signals, and all of these cell types were increased in number upon challenge. CD64 is the FcγR1 receptor, which binds to immune complexes comprising IgG-antigen complexes and which can induce CD4+ and CD8+ T-cell activation when expressed on monocyte-derived dendritic cells (Schuurhuis et al., 2002; Tanaka et al., 2009; Uo et al., 2013). CD1a has been known for decades as a phenotypic marker of human epidermal Langerhans cells (LC). Like the other members of the CD1 family, CD1a displays lipids; however, unlike the other members, presentation to T-cells evokes the release of IL-22, IL-13 and IFNγ from T-cells. The role of CD1a macrophages and monocytes in UC has yet to be examined as it is unclear whether this effect is beneficial or further fuels inflammation.

Finally, TSLPR is thought to mediate signals released from epithelial cells that secrete TSLP in response to damage, thereby activating memory T-cells and natural killer T-cells to release Th2-type cytokines and monocyte-derived dendritic cells to promote a Th2 response, resulting in a healing process (Soumelis et al., 2002; Nagata et al., 2007; Ito et al., 2012). This idea is supported by the observed increase of HGF, TGFβ1 and TARC expression, all of which are important factors in wound healing processes. The fact that both CD64+ and CD1a+ monocytes have the potential to induce autoimmune responses that cause damage to epithelial cells and the fact that activation of CD8+ cells was observed upon challenge with ethanol suggests that the observed inflammation might be driven by an ‘autoimmune’ response, meaning that the aspect of autoimmunity in UC might be examined and addressed in this model. As one of the susceptibility loci for UC is the gene encoding IL-10, loss of tolerance has been considered as a driver of the disease for some time (McGovern et al., 2010). This idea is also corroborated by various animal models in which either loss of IL-10 induces spontaneous colitis or application of IL-10 ameliorates disease symptoms (Kuhn et al., 1993; Steidler et al., 2000). However, it has been thought that commensal bacteria, which are immunologically silent under healthy conditions, were the preferred targets of the autoimmune response. Recently, other autoreactive antibodies, such as perinuclear anti-neutrophil cytoplasmic antibodies (pANCAs) and those that recognize goblet cells and granulocyte macrophage colony-stimulating factor have been detected in UC individuals, expanding the view of autoimmunity in UC (Seibold et al., 1998; Kovacs et al., 2012; Dabritz et al., 2013). Whether these autoantibodies are causative for or are the result of ongoing inflammation, for sometimes decades, remains to be determined.

CD14+ CD163+ monocytes are sometimes considered as counter players of CD14+ CD64+ monocytes because they play a crucial role in resolution of inflammation. The fact that both frequencies of both subtypes are elevated might indicate that resolution of inflammation is an intrinsic part of the inflammatory response.

The fact that TGFβ1, TARC and HGF expression levels increase upon challenge with ethanol strongly supports our hypothesis that part of the inflammatory response are wound healing processes to protect the damaged epithelia. Increased HGF expression corroborated the observation of epithelial proliferation in histological sections from ethanol-challenged mice.

Although below detection level in most challenged mice, the expression of IFNγ could be verified, especially in mice that had been treated with pitrakinra. Suppression of wound healing processes most probably results in increased inflammation, reflected by the expression of human IFNγ and human TNFα.

### Treatment with infliximab

Ethanol-challenged mice responded to treatment with the anti-TNFα monoclonal antibody infliximab, which has become the preferred therapeutic in severe cases of UC. Both, the clinical activity and the histological score declined in mice that had been treated with infliximab. Most probably, infliximab not only exerts its activity by trapping soluble TNFα but also by binding to surface-bound TNFα that is expressed by T-cells, macrophages and monocytes, thereby inducing apoptosis through the IgG1 effector function (Scallon et al., 1995). Analysis of human leucocytes that had been isolated from mice treated with infliximab corroborated this assumption. Treatment with infliximab resulted in a decline of antigen-experienced CD4+ cells and CD14+ monocytes bearing CD64, CD1a and CD163, all of which were increased in response to challenge with ethanol. As observed in UC individuals, treatment with infliximab had opposing effects on HGF, TARC and TGFβ1 expression (Ulrich Mansmann and our unpublished data). Infliximab reduced HGF and TARC expression, and supported TGFβ1 expression, albeit the effect on TGFβ1 expression in mice was not as profound as that in humans.

The mechanism by which infliximab exerts its efficacy might be explained by the two inflammatory responses prevailing in UC-affected humans. Here, an acute response was defined by the presence of immune cells of the adaptive immunity, CD11b+ macrophages and elevated expression of HGF and TARC. Treatment with blockers of TNFα led to the suppression of this acute inflammation and favored the remodeling inflammatory condition characterized by CD14+ monocytes, natural killer T-cells, and elevated expression of TGFβ1 and periostin (Ulrich Mansmann and our unpublished data). Although this profile was not completely reflected in the mouse model, the data suggest an impairment of the acute arm of inflammation by infliximab while leaving the remodeling arm unaffected.

### Treatment with pitrakinra

Rather unexpectedly, treatment with pitrakinra exacerbated the inflammatory response. The clinical activity and the histological score did not decline, and visual inspection of histological sections revealed an ongoing severe inflammation. Analysis of human leucocytes that had been isolated from spleens might give an explanation. Treatment with pitrakinra resulted in an increase of CD3+, CD8+ and central memory CD8+ T-cells, along with CCR4-expressing CD11b+ macrophages and CD14+ monocytes. In contrast, subtypes of CD14+ monocytes, which are supposed to express IL-4Rα1, declined, and this was accompanied by decreased expression of TGFβ1 and HGF. Conversely, TARC expression levels were increased. These results might also be explained by the identified inflammatory conditions in UC-affected individuals (Ulrich Mansmann and our unpublished data). Pitrakinra might impair the wound healing arm of inflammation that is driven by a Th2-characterized inflammatory environment, thus favoring the acute arm that is characterized by activated CD8+ cells. This assumption is corroborated by previous findings that relate fibrogenesis to IL-13 (Fichtner-Feigl et al., 2007). The futility of addressing this arm of inflammation by suppressing Th2 responses was also corroborated by a clinical phase II trial using anrukinzumab. Treatment with the anti-IL-13 antibody had no effect on the clinical score (Reinisch et al., 2015). The idea that pitrakinra favors the acute inflammatory condition is also supported by the increased expression of TARC and decreased expression of TGFβ1. Both were found to be associated with acute and remodeling inflammatory conditions in UC-affected individuals (Ulrich Mansmann and our unpublished data).

Thus, how reflective is this model of the human disease? Obviously, one cannot expect to cover all aspects of this complex and highly dynamic disease. Manifestations of the disease are extremely diverse and might have different causes, all of which are covered by the umbrella diagnosis of UC. One also has to keep in mind that, in this model, inflammatory responses are restricted to PBMCs, which do not represent the entire repertoire of inflammatory cells. However, as discussed for UC, monocytes and macrophages play a crucial role in the mouse model, either fueling the inflammatory response or guiding it towards wound healing processes. In this model, cell types were detected that have been previously identified as being crucial to pathology in UC patients. This includes CD1a- and CD64-expressing CD14+ monocytes, the frequencies of which are found to be elevated in the colon of UC individuals as compared to those in non-UC subjects (Ulrich Mansmann and our unpublished data), and which were found to be increased in the human leucocyte cell population isolated from murine spleens that had been challenged with ethanol. Furthermore, we could detect the same cells – CD1a-, TSLPR-, and CD80- and/or CD86-expressing macrophages; and CD1a-, and CD80- and/or CD86-expressing monocytes – in the murine colon, which were also present in the colon of UC individuals. In addition, HGF, TARC and TGFβ1, which have been associated with inflammatory conditions identified in UC individuals, were also induced in this model. Treatment of mice with infliximab reflected responses observed in UC individuals treated with blockers of TNFα, as shown by the decreased expression of HGF and TARC and by the increased or unaffected TGFβ1 expression. There are, however, differences when it comes to the role of CD11b+ macrophages, which play a crucial role in the acute inflammatory condition in humans as opposed to inflammation in mice, which seemed to be governed by CD14+ macrophages. In addition, in humans, CD4+ and CD8+ cells are prominent, whereas inflammatory cell populations were dominated by CD14+ and CD11b+ macrophages in mice. Both effects could be ascribed to the rather short time after reconstitution. Studies allowing for longer times of engraftment might shift the balance of cell types.

Although ethanol is considered a dietary factor in UC (Jowett et al., 2004), by no means can we consider rectal application of 50% ethanol a naturally occurring trigger of relapses in UC. So far, we can only speculate how ethanol induced inflammation in our mouse model. Ethanol or its metabolites might act as toxins to cause inflammation and exuberant fibrosis, as known from liver diseases. It might also cause a temporal breach of the epithelial barrier, allowing the penetration of bacteria to induce inflammation. Alternatively, ethanol might denature proteins to support an immunological reaction. As shown in a previous study, oxazolone is toxic in NSG mice in the absence of PBMCs; ethanol is also toxic, albeit to a lesser extent than oxazolone. Mice challenged with oxazolone died spontaneously within 4-12 h post challenge as opposed to ethanol-challenged mice, which had to be euthanatized at 48 h post challenge. The toxic effect of ethanol was proven by analysis of the macroscopic appearance of the colon, which displayed constipation and the beginning of necrosis. Histological analysis revealed edema and an influx of neutrophils, indicating either influx of bacteria as a result of the barrier breach or wound healing as a result of epithelial cell damage, or a combination of both. The fact that NSG mice exhibited lower expression of mucins, as shown by PAS staining, might give an explanation for the high susceptibility of NSG mice to ethanol. Mucins provide a protective shield to impair the contact of bacteria with the mucosa. Alternatively, the hydrophilic mucus might bind to ethanol or oxazolone, thus preventing the contact of these toxins with the mucosa. However, how reconstituted PBMCs can possibly mitigate the toxic effects of ethanol and, as previously observed, those of oxazolone remains elusive and has to be elucidated in future studies. As in previous studies, the immunological background of the donor influences inflammatory responses (Nolte et al., 2013a,b; Zadeh-Khorasani et al., 2013). As shown in this study, NSG mice that had been reconstituted with PBMCs from a healthy donor were affected by the application of ethanol; however, this resulted most probably from ethanol intoxication. These mice did not develop diarrhea, and colon appearance and histology were normal. The increase in CD64+ CD14+ monocytes in response to challenge that was observed in mice reconstituted with PBMCs from UC individuals might provide one explanation. Autoantibody-autoantigen immune complexes present in the UC background might activate the FcγR1 (CD64) receptor. (Uo et al., 2013). Therefore, we think that this model might reflect the autoimmune aspect of the disease. Future studies have to show whether colitis-like symptoms can also be evoked through exposure to autoantigens, such as proteinase 3 (PR3) detected by pANCAs, which have been identified as a biological marker of UC (Arias-Loste et al., 2013). This effect has been previously shown for CD4+ cells monospecific to ovalbumin (Yoshida et al., 2001). Regardless of the trigger, we feel that this model can be used to dissect and examine different arms of the inflammation using inhibitors addressing human target molecules.

In summary, we have shown that this model is partially reflective of the human disease and can be used to study the efficacy of therapeutics addressing human target molecules on immune cells. In combination with immune profiling of UC-affected individuals and selection of subgroups of individuals for reconstitution of mice, it might also improve the translatability of preclinical animal studies to future clinical studies. We feel confident that it might be used to elucidate the cellular mechanisms that induce and sustain flares of this disease of subjects in an *in vivo* model. Finally, it might be a useful surrogate model for non-human primates, which are used when high-sequence homology and cross reactivity of human proteins are necessary.

## MATERIALS AND METHODS

### Ethical considerations

All donors gave informed written consent, and the study was approved by the Institutional Review Board (IRB) of the Medical Faculty at the University of Munich (2015-22).

Animal studies were approved by the ethics committee of the government of Upper Bavaria, Germany (55.2-1-54-2532-65-11 and 55.2-1-54-2532-76-15) and performed in compliance with German animal welfare laws.

### Isolation of PBMCs and engraftment

Peripheral blood was collected from the arm vein of UC-affected individuals. Approximately 60 ml of blood in trisodium citrate solution (S-Monovette, Sarstedt, Nürnberg, Germany) was diluted with Hank's balanced salt solution (HBSS; Sigma-Aldrich, Deisenhofen, Germany) in a 1:2 ratio, and 30 ml of the suspension was loaded onto Leukosept tubes (Greiner Bio One, Frickenhausen, Germany). Cells were separated by centrifugation at 400 ***g*** for 30 min and no acceleration. The interphase fraction containing PBMCs was extracted and diluted with PBS to a final volume of 40 ml. Cells were counted and centrifuged at 1400 ***g*** for 5 min. The cell pellet was resuspended in PBS at a concentration of 4×10^6^ cells in 100 µl.

Six- to twelve-week-old NOD-*scid* IL-2Rγ^null^ mice were engrafted with 100 µl cell solution into the tail vein on day 1.

### Study protocol

NOD.cg-Prkdc^SCID^ Il2rg^tm1Wjl^/Szj mice (abbreviated as NOD-*scid* IL-2Rγ^null^) were obtained from Charles River Laboratories (Sulzfeld, Germany). Mice were kept under specific pathogen-free conditions in individually ventilated cages. The facility was controlled according to the Federation of Laboratory Animal Science Association (FELASA) guidelines. Following engraftment (day 1), mice were pre-sensitized through rectal application of 150 µl of 10% ethanol on day 8 using a 1-mm cat catheter (Henry Schein, Hamburg, Germany). The catheter was lubricated with Xylocain^©^ Gel 2% (AstraZeneca, Wedel). Rectal application was performed under general anesthesia using 4% isoflurane. Post application, mice were kept at an angle of 30° to avoid ethanol dripping. On days 15 and 18, mice were challenged with rectal application of 50% ethanol following the protocol described for day 8. Mice were killed on day 21. Pitrakinra (10 µg in 0.5% methylcellulose, 0.05% Tween-80) in PBS (Zadeh-Khorasani et al., 2013) was applied on days 7-9 and 14-21. In these groups, sterile saline (B. Braun Melsungen AG, Germany) served as control. Infliximab [6 mg/kg (Remicade^©^, Janssen, The Netherlands)] was applied on days 7, 14 and 17. An isotype antibody (human IgG1, kindly provided by, MorphoSys AG) was used as control. All treatments were applied intraperitoneally.

### Clinical activity score

Assessment of colitis severity was performed daily according to the following scoring system. The loss of body weight was scored as follows: 0% (0), 0-5% (1), 5-10% (2), 10-15% (3), 15-20% (4). The stool consistency was scored as follows: formed pellet (0), loose stool or unformed pellet (2), liquid stools (4). Behavior was scored as follows: normal (0), reduced activity (1), apathy (4) and ruffled fur (1). Posture was scored as follows: intermediately hunched posture (1), permanently hunched posture (2). The scores for each criterion were added daily into a total score with a maximum of 12 points per day. Animals who suffered from weight loss >20%, rectal bleeding, rectal prolapse, self-isolation or a severity score >7 were killed immediately and not taken into count. For statistical analysis, all scores over all days were added to give the final score.

### Isolation of human leucocytes

For isolation of human leucocytes from murine spleen, spleens were minced and cells filtrated through a 70-µl cell strainer followed by centrifugation at 1400 ***g*** for 5 min and resuspension in FACS buffer. Cell suspensions were filtrated one more time using a 35-µm cell strainer for further purification before labeling the cells for flow cytometry analysis.

For isolation of lamina propria mononuclear cells (LPMCs), a protocol of Weigmann et al. (2007) was modified and used. The washed and minced colon was predigested twice for 20 min each time in pre-digestion solution containing 1× HBSS (Thermo Scientific, Darmstadt, Germany), 5 mM EDTA, 5% FCS, 100 U/ml penicillin-streptomycin (Sigma-Aldrich, St. Louis, MO) in an orbital shaker with slow rotation (40 ***g***) at 37°C. Epithelial cells were removed by filtering through a nylon filter. Following washing with RPMI, the remaining colon pieces were digested twice for 20 min each time in digestion solution containing 1× RPMI (Thermo Scientific, Darmstadt, Germany), 10% FCS, 1 mg/ml collagenase A (Sigma-Aldrich, St. Louis, MO), 10 KU/ml DNase I (Sigma-Aldrich, St. Louis, MO), 100 U/ml penicillin-streptomycin (Sigma-Aldrich, St. Louis, MO) in an orbital shaker with slow rotation (40 ***g***) at 37°C (Weigmann et al., 2007).

Isolated LPMCs were collected by centrifugation at 500 ***g*** for 10 min and resuspended for FACS analysis. Cell suspensions were filtrated one more time using a 35-µm cell strainer for further purification before labeling the cells for flow cytometry analysis.

### Flow cytometry analysis

Human leucocytes were stained with the antibodies described in Table S4. Antibodies were diluted 1:200, and 100 μl was used to stain 10^6^ cells.

All antibodies were purchased from BioLegend (San Diego, USA) and used according to the manufacturer's instructions. Samples were measured using a BD FACS Canto II™ instrument and analyzed with FlowJo 10.1-Software (FlowJo LLC, OR).

### Histological analysis

Distal parts of the colon were fixed in 4% formaldehyde for 24 h, followed by 70% ethanol, and were routinely embedded in paraffin. Samples were cut into 3-µm sections and stained with hematoxylin and eosin (H&E). Epithelial erosions were scored as follows: no lesions (1), focal lesions (2), multifocal lesions (3) and major damage with involvement of basal membrane (4). Inflammation was scored as follows: infiltration of a few inflammatory cells into the lamina propria (1); major infiltration of inflammatory cells into the lamina propria (2); confluent infiltration of inflammatory cells into the lamina propria (3); and infiltration of inflammatory cells, including tunica muscularis (4). Fibrosis was scored as follows: focal fibrosis (1), multifocal fibrosis and crypt atrophy (2). The presence of edema, hyperemia and crypt abscesses was scored with one additional point in each case. The scores for each criterion were added into a total score ranging from 0 to 12. Sections were scored by a certified veterinarian pathologist in a blinded manner. To evaluate the distribution of goblet cells, sections were stained with PAS. Images were taken with a Zeiss AxioVert 40 CFL camera. Figures show representative longitudinal sections at the original magnification. In Adobe Photoshop CS6, tonal correction was used in order to enhance contrast within the pictures and was applied equally to the whole image.

### RNA analysis

#### RNA extraction and cDNA synthesis

Approximately 1-cm (in length) samples from distal parts of the colon were disrupted and homogenized with the TissueLyser LT (Qiagen, Hilden, Germany) followed by total RNA extraction according to the manufacturer's instruction using RNeasy Plus Universal Mini Kit (Qiagen, Hilden, Germany) and chloroform (Sigma-Aldrich, St. Louis, MO). No further treatment with DNase was needed because gDNA Eliminator Solution is included in the kit.

For cDNA synthesis, 5 μg of total RNA was used. Reverse transcription was performed in a Mastercycler gradient (Eppendorf, Hamburg, Germany) using QuantiNova Reverse Transcription kit (Qiagen, Hilden, Germany). Samples were diluted with RNase-free water to obtain a cDNA concentration between 10 pg and 100 ng as required by the TaqMan Fast Advanced Master Mix protocol (Thermo Fisher Scientific, Waltham, MA).

RNA and cDNA purity was assessed using a Nanodrop 2000 spectrophotometer (Thermo Fisher Scientific, Waltham, MA).

#### Quantitative RT-PCR

According to the TaqMan Fast Advanced Master Mix protocol (Thermo Fisher Scientific, Waltham, MA) quantitative RT-PCR was performed using the Applied Biosystems StepOnePlus RT-PCR system (Thermo Fisher Scientific, Waltham, MA). Single-tube TaqMan gene expression assays (Thermo Fisher Scientific, Waltham, MA) included the housekeeping genes GAPDH (Mm99999915_g1) and GUSB (Mm00446953_m1), as well as TGFβ (Mm 01178820_m1), HGF (Hs04329698_m1), CCL17 (Mm01244826_g1), IFNγ (HS00989291_m1) and TNFα (HS01113624_g1) (Thermo Fisher Scientific assay IDs are given in brackets). Analysis was performed using StepOnePlus™ Software v2.3. For expression analysis, a mean value of cycle threshold values was calculated for two housekeeping genes. Relative expression values for the respective analyzed genes were calculated as the difference between the mean cycle threshold (CT) value of the housekeeping genes and the respective analyzed gene (delta CT). Relative expression is depicted as the logarithmic value of the delta CT.

### Statistical analysis

Statistical analysis was performed with R software: a language and environment for statistical computing (R Foundation for Statistical Computing, Vienna, Austria; https://www.R-project.org/). Variables are represented with mean, standard deviation, median and interquartile range (IQR) values. A two-sided Student's *t*-test and confidence level=0.95 was used to compare binary groups, and for more than two groups, ANOVA followed by Tukey's honest significant difference (HSD) was conducted. Sample size calculations were assessed based on a confidence interval of 95% and a power of 80%.
